# Mini-review: Strategies for Variation and Evolution of Bacterial Antigens

**DOI:** 10.1016/j.csbj.2015.07.002

**Published:** 2015-07-26

**Authors:** Janet Foley

**Affiliations:** 1320 Tupper Hall, Veterinary Medicine and Epidemiology, UC Davis, Davis, CA 95616, United States

**Keywords:** Homologous recombination, Hypermutability, Immune evasion, Pseudogene

## Abstract

Across the eubacteria, antigenic variation has emerged as a strategy to evade host immunity. However, phenotypic variation in some of these antigens also allows the bacteria to exploit variable host niches as well. The specific mechanisms are not shared-derived characters although there is considerable convergent evolution and numerous commonalities reflecting considerations of natural selection and biochemical restraints. Unlike in viruses, mechanisms of antigenic variation in most bacteria involve larger DNA movement such as gene conversion or DNA rearrangement, although some antigens vary due to point mutations or modified transcriptional regulation. The convergent evolution that promotes antigenic variation integrates various evolutionary forces: these include mutations underlying variant production; drift which could remove alleles especially early in infection or during life history phases in arthropod vectors (when the bacterial population size goes through a bottleneck); selection not only for any particular variant but also for the mechanism for the *production* of variants (i.e., selection for mutability); and overcoming negative selection against variant production. This review highlights the complexities of drivers of antigenic variation, in particular extending evaluation beyond the commonly cited theory of immune evasion. A deeper understanding of the diversity of purpose and mechanisms of antigenic variation in bacteria will contribute to greater insight into bacterial pathogenesis, ecology and coevolution with hosts.

## 1 Introduction

The survival of a pathogen requires evasion of host immunity, either by switching hosts, becoming free-living some of the time, finding sites within a host that are “privileged” or hidden from host immunity, or switching the composition of an antigen. Pathogens commonly interact with hosts through proteins or other macromolecules on their cell surface; those that incite immune response from the host are designated as antigens. Phenotypic variation is subject to evolution if that variation allows these molecules the pathogen to evade host immunity or perform some other function, e.g., utilize diverse host niches. The ability to vary antigen structure has evolved independently multiple times across kingdoms and phyla of eukaryotes and prokaryotes, reaching particular importance in obligately parasitic organisms that cause chronic infection in hosts. For example, because vector-borne and sexually transmitted pathogens often rely for survival on infection that persists long enough for a new vector or host to acquire the infection, chronic infection and antigenic variation are common in these pathogens [1]. Mechanisms of production of variant antigen genes vary in specifics, but for most eukaryotes and prokaryotes (in contrast with viruses, which often utilize point mutations), larger scale variation is the rule [1], well reviewed in [2]. There are epigenetic, transcriptional, and translational regulatory mechanisms for varying antigens as well, which are outside the scope of the present paper. The bacteria span alpha-Proteobacteria in the order Rickettsiales (*Anaplasma*, *Ehrlichia*, and *Wolbachia* genera); beta-Proteobacteria in the order Neisseriales; *Treponema* and *Borrelia* species in the phylum Spirochaetes, family Spirochaetacea, and even Mollicutes (*Mycoplasma* spp., phylum Terericutes). This review will describe a suite of prokaryotic pathogens that vary antigens as part of a strategy to evade host immunity and promote chronic infection. Terminology will be used consistent with the literature associated with each pathogen, but will be defined. I describe mechanisms for production of variants, and synthesize evolutionary forces and constraints that shape the coevolutionary phenomenon of antigenic variation among diverse bacterial genera.

### 1.1 *Anaplasma phagocytophilum* and *Anaplasma marginale*

An extensive body of literature evaluates antigenic variation in the Anaplasmataceae, reviewed earlier [3]. Here we update literature with some recent findings and synthesis, broadening the discussion to other members of the family. *A. phagocytophilum* is an obligately parasitic, intra-neutrophilic, tick-transmitted bacterial pathogen that causes granulocytic anaplasmosis (GA) of humans and other animals. Over the course of infection, hosts acquire temporary resistance to GA as they develop immunity specific for the major antigen, major surface protein 2 (Msp2). The function of this protein is not completely known. The Msp2 forms multimeric complexes in the bacterial membrane and then, on the human neutrophil, Msp2 cooperatively binds α1,3 fucosyl transferases that construct the sialyl Lewis ^X^ (which typically caps the P-selectin glycoprotein ligand-1) [4], steps necessary for bacterial internalization into cell. As hosts develop antibodies against expressed Msp2 variants, the bacterium then utilizes Rec-F dependent mechanisms to recombine “functional pseudogenes” of the pfam01617 family (common within Anaplasmataceae) sequentially into a hypervariable region of an “expression cassette” with conserved 5′ and 3′ ends. The expression cassette refers to the single locus in the genome where a full-length coding sequence and promoter serve for transcription of an RNA for the surface protein. Variation in the expression cassette presumably bypasses host immunity and contributes in some host species to persistent infection [5,6]. Indeed, antibodies produced against specific Msp2 variants diminish rapidly as new variants emerge, consistent with antigenic variation as an immune evasion strategy [7].

In the case of *A. phagocytophilum*, pseudogenes are considered functional in that they serve as donor DNA into a functional expression cassette. Approximately 100 pseudogenes are present in the original fully sequenced genome, human-origin HZ, representing the possibility for almost unlimited antigenic diversity [8]. This is remarkable given the small genome size of only 1.4 Mb [8]. Of these pseudogenes, most have high identity to some expression cassette that has been sequenced from a naturally occurring host, suggesting that they have been used as a recombination donor [9]. Interestingly, high matches of HZ pseudogenes occurred for predominantly North American, canine, and human hosts, possibly reflecting the origin of the genome from which these pseudogenes were sequenced [9]. In other words, if the full genome from which pseudogenes were being evaluated was a European or cervid strain, it is possible that higher matches would be to European or ungulate expression cassette.

Pseudogenes with high identity to naturally occurring expression cassettes among diverse hosts showed some clustering on a phylogenetic tree and in space on the genome, although many pseudogenes did not fit in convenient clusters [9]. There was some evidence for multiple tandem repeats of pseudogenes but these did not cluster on the phylogenetic tree, suggesting that they were not derived from local duplications. Pseudogenes near the putative origin of replication were more likely to match highly to some host expression cassette. All of these data suggest a phenotype of hyper-recombination with natural selection retaining pseudogenes in the genome, in particular locations.

*A. marginale*, a related pathogen that infects red blood cells and causes anemia of deer and cattle, uses a similar recombination mechanism to insert pseudogene fragments into a single polycistronic expression cassette with a central hypervariable region for its Msp2, although with some important differences compared to *A. phagocytophilum* [10]. In contrast to *A. phagocytophilum*, there are only 5–7 pseudogenes in the *A. marginale* St. Marie's genome, distributed across the genome [11,12]. In fact, depending on the strain, some of the pseudogenes are duplicates or are so truncated that their role in gene conversion is unclear, and the fragments that are actually involved in protein switching have been called “donor alleles” [13]. There are several different mechanisms by which variants are produced, such as an “anchored gene conversion” in which part of a recombination complex is inserted into an invariant region and another into a hypervariable region; small insertions within the hypervariable region; or scattered substitutions across the region [11,13]. In part because mosaics of the expression cassette can be constructed through segmental recombination, very high diversity can occur in an approximately 100 amino acid antigenically variable domain [13]. High and comparable levels of diversity in *msp2* alleles occur among strains and among pseudogenes within each individual strain, which was interpreted to indicate that there is selection within host individuals and host populations to retain multiple alleles [14]. There is strong evidence that the production of novel Msp2 proteins contributes to the evasion of host immunity. Rickettsemia in cattle is highly cyclical with levels varying from 10^2^/ml to 10^7^/ml over 6 week intervals, ultimately persisting for years [15,16]. The central hypervariable region of *msp2* encodes B-cell epitopes and *msp2* variants emerge shortly after defined immunoglobulins are produced that recognize an older Msp2 variant [15,17]. Superinfection (i.e., infection with a second strain in already rickettsemic animal) is possible as long as the new strain has at least one unique donor allele [14]. The *msp3* gene family of *A. marginale* also is polymorphic [18] and the simultaneous switching of Msp2 and Msp3 is proposed to extend immune evasion considerable [17].

### 1.2 *Ehrlichia* Spp.

The genus *Ehrlichia*, after some recent taxonomic reclassification, includes the closely related species *Ehrlichia chaffeensis* and *Ehrlichia ewingii* which cause monocytic and granulocytic ehrlichiosis, respectively, in humans, *Ehrlichia canis* which causes monocytic ehrlichiosis primarily in dogs, and *Ehrlichia ruminantium* which causes heartwater in ungulates. These obligately intra-cellular and tick-transmitted bacteria cluster together in the family Anaplasmataceae with members of the *Anaplasma* genus. Like *Anaplasma* spp., *Ehrlichia* spp. bacteria have an immunodominant, surface-exposed, varying antigens with both conserved and variable regions, named MAP-1 for *E. ruminantium*, P28 for *E. chaffeensis*, and p28/p30 for *E. canis*. Recombinant P28 protein confers immunity to *E. chaffeensis* challenge in a mouse model [19] and a MAP-1 DNA vaccine protects mice from *E. ruminantium* [20].

There appear to be important differences in the multiple-gene family that encodes these *Ehrlichia* spp. antigens, compared with that in *Anaplasma* and related species. In *Ehrlichia* spp., this family is fairly small and consists of 17–22 tandemly arranged, mostly complete genes or gene fragments with functional promoters and 200–600 bp intergenic spaces [19–23]. Statistical and genetic analysis of *E. ruminantium* full genomes indicated that the *map-1* genes had evidence of a very high rate of synonymous substitutions per site, consistent with past, frequent homologous recombination [24,25]. Positive selection maintaining *map-1* variants was indicated by one analysis [26], but not supported in another [25]. However, unlike diversity generated by gene conversion in *Anaplasma* spp., the expressed diversity of the *Ehrlichia* sp. antigen may be due to differential expression of intact gene copies, as shown for *E. chaffeensis* and *E. canis* [21,27]. Transcription at the 5′ end of the *E. canis omp* gene cluster appears to be polycistronic, but monocistronic at the 3′ end [21]. Interestingly, even though *E. canis* occurs across most of the world, many genes are relatively conserved, even including the *p28/p30* genes [28]. There are other major antigens, particularly the secreted gp36 protein which elicits strong antibody responses, is highly variable across strains, and is coded for by a gene with 11–18 tandem repeats [28].

### 1.3 *Wolbachia* Spp.

Wolbachias are parasitic and commensal rickettsial bacteria of invertebrates including insects, chelicerates, crustaceans, and nematodes. Although *Wolbachia* usually is vertically transmitted, there are cases of horizontal transmission even across host species [29]. *Wolbachia* infection commonly causes reproductive disorders such as feminization, parthenogenesis, and cytoplasmic incompatibility, but also direct morbidity such as neural tissue invasion and destruction [30]. The species is divided phylogenetically into several supergroups but there is evidence for extensive horizontal transfer due to recombination facilitated by phages, IS elements, and other mechanisms [31]. Wolbachias have a major surface protein Wsp; the *wsp* gene is homologous to those from related rickettsias [32]. The gene has four conserved transmembrane regions interspersed with hypervariable regions (HVRs). Comparative analysis of 40 *wsp* genes indicated multiple single nucleotide polymorphisms but also separate recombination events as the source of variability for each of the HVRs [33]. Recombination events appeared to have occurred both within and among supergroups.

Importantly, the variability in Wsp highlights the problem with inferring that “antigenic variation” evolves to escape immunity, because Wolbachias primarily infect invertebrate organisms that lack adaptive immunity. *Wolbachia* Wsp is under purifying selection, based on analysis of rates of synonymous vs. nonsynonymous substitutions, although there also are discrete regions corresponding to the HVRs within the gene apparently undergoing strong positive selection [34]. Interestingly, positive selection was not detected in mutualistic wolbachias from nematodes and the positive selection detected in parasitic wolbachias was concentrated outside the predicted transmembrane region of the protein [26]. *wsp* paralogs designated *wspB* and *wspC* were detected in the *w*Mel *Wolbachia* genome but they did not appear to be donors for the HVRs and *Wolbachia wsp* HVRs were not homologous with genes or fragments from other bacteria [33]. The positive selection regime on *wsp* of parasitic wolbachias must be variable. In vertebrate-pathogenic bacteria, antigens vary in concert with varying *adaptive* immunities and this variable selection regime maintains genetic variation in the pathogen. However invertebrates are thought to lack adaptive immunity, which raises the question whether the *innate* immunity which arthropods do have might vary in a way that could impose selection to maintain *wsp* variation. In fact a study in *Drosophila* showed precisely the type of adaptive immunity which could exert such selection [35]. In that study, fly immunity was specific to *Streptococcus pneumoniae*, was long-lasting and capable of preventing further infections, and derived from priming of phagocytes. How general such adaptive immunity is in arthropods and whether it is strong enough to produce the variable signal seen in *wsp* are unknown. However, *wsp* has another purpose—it is a host cell attachment protein: even in the absence of immune pressure, the mosaic gene may have evolved for occupation of different host niches. Less probably, variable *wsp* may be a byproduct of the hyper-recombination that occurs in the *Wolbachia* genome in general.

### 1.4 Relapsing Fever *Borrelia* Spp.

Relapsing fever is a potentially severe disease caused by several related *Borrelia* species in North America and Africa and is transmitted by various Argasid (soft) ticks or lice to humans and/or other animals. As the name implies, the clinical signs wax and wane, often becoming severe for several consecutive nights. It is thought that the interaction of host immunity and bacterial immune evasion accounts for this relapsing phenomenon. Antibody production alone may suffice to clear a relapsing fever infection, suggesting that humoral immunity is the main driving force for these *Borrelia* spp. to vary antigens [36], and the antibody class IgM [37]. The rate of antigen switching is about 10^− 4^ to 10^− 2^ per cell per generation while in the vertebrate host [38,39] and, during the course of infection, the emergence of new serotypes follows a “loose order” [40]. These bacteria utilize multiple mechanisms of switching antigens, including gene conversion, accumulation of point mutations, DNA rearrangement, and transcript regulation.

The relapsing fever *Borrelia* spp. have an unusual collection of linear chromosomes (sometimes described as a single chromosome with multiple linear plasmids) and two subtelomeric genes coding for antigens, the *vlp* and *vsp* genes (variable long and short proteins, respectively, together referred to as *vmp* for variable major protein). Throughout the genome (chromosome plus plasmids), each of these genes contains about 30 copies [41]. An important mechanism for the production of variant Vmps is duplicative unidirectional, nonreciprocal transfer of nucleotides from a donor allele into the expression site downstream of a promoter [42], in which an upstream homology sequence (UHS) at the 5′ end of the *vmp* and the downstream homology sequence (DHS) serve as sites of initiation of gene conversion [43]. Other factors that contribute to the process include the sigma-70 type promoter, the ribosome binding site, an upstream long string of T residues that enhances transcription, and three imperfect inverted repeats [44,45]. Inverted repeat sequences in the DHS probably help promote recombination [46]. Rarely, hybrid *vmp* genes may occur due to partial length gene conversion but are short-lived in the face of host immunity [47] and there appears also to be formation of mosaics among archived alleles [48]. A second mechanism for the generation of new variants is via gene deletion between a pseudogene and expression site within a single linear plasmid [49]. The *vmp7* gene is linked to a pseudogene for *vmp26* near the telomere of a linear plasmid. Gene conversion within the expression site occurs at the sites of a 20 nucleotide fragment common to both the *vmp7* gene and *vmp26* pseudogene, deleting most of the expressed *vmp7*, and positioning the pseudogene immediately downstream of the first two codons from the *vmp7*, thereby allowing the *vmp26* to be expressed.

A single infected individual typically has a set of bacteria expressing a single Vmp population, with only rare variants present at any given time [38]. In the tick vector, a *vtp* gene expresses a protein needed for tick infectivity (Vtp for variable tick protein), and typically *vtp* and *vmp* expression are toggled on and off during tick and host infection [50]. However, if *vmp* is inactivated and Vtp continues to be expressed, experimental mice may be infected but infection fails to persist, as IgM targets the Vtp and eliminates the infection [51]. Statistical analysis of repeats associated with varying antigens supported the role of strong natural selection maintaining rare alleles in the population [48]. It is likely that the balancing selection that maintains this diversity is imposed by evolving host immunity, but utilization of multiple host niches also could drive some of the retention of diversity, given the documented association of some *vspA* expressed variants of *Borrelia turicatae* with various tissue tropisms (e.g., neurologic tissue) [52]. The pattern of occurrence of the variants relates to the relationships of the silent allele with the extragenic sequence elements near the donor locus and the expression site [53]. Greater homology between donor alleles and the expression site did *not* result in higher probability of recombination. In contrast, variants are more likely to be expressed if they are highly homologous with the UHS at the 5′ end of the *vmp* and closer to the DHS.

### 1.5 Lyme Borreliosis *Borrelia* Spp.

Lyme borreliosis (caused by *Borrelia burgdorferi* sensu lato) is the most common vector-borne disease in North America and Europe [54]. The bacteria occur across the Holarctic and are transmitted by *Ixodes* spp. ticks [55]. Acute disease is characterized by an early localized rash at the tick bite site (erythema migrans) sometimes followed by extracutaneous, potentially chronic disease including fever, fatigue, headache, and arthralgia, with possible oligoarthritis, carditis, lymphocytic meningitis, and peripheral neuropathy [56]. *B. burgdorferi* has multiple antigens anchored to the outer membrane, although most do not vary [57]. Despite homology with the *Borrelia hermsii* surface Vmp protein, the OspC protein, which is expressed as *B. burgdorferi* transits the tick salivary glands to the host, does not have within-cell variants that can be expressed sequentially during host infection. Rather, persistence in the host occurs in concert with production of variants at the *vls* locus.

The *vls* expression site (*vlsE*) is located near the end of the 20 kbase linear lp28-1 plasmid, contiguous with 15 unexpressed cassettes with high homology to the *vlsE* [58]. The *vlsE* locus has a central variable region consisting of six variant and six invariant regions, surrounded by conserved sequence; each of the cassettes also has six variable regions and conserved 5′ and 3′ ends [58]. The antigen is the 35 kDa lipoprotein VlsE of which variant regions are exposed to host antibodies while invariant regions maintain overall protein structure and function [59]. Little else is known of the function of VlsE, except that it undergoes antigenic variation and levels of expression appear to vary depending on what host tissue is infected [60]. Variants are produced through gene conversion via recombination between the *vlsE* expression site and any of the 15 cassettes [61]. Independent disruption of 17 genes involved in DNA recombinant and experimental infection with the mutants documented a crucial role of ruvAB Holliday branch migrase, encoded by *ruvA* and *ruvB*, in producing *vlsE* variants [62]. In contrast to relapsing fever *Borrelia* spp., recombinants in *B. burgdorferi* are not constrained by conserved upstream and downstream homology sequences. Short (as little as one bp) or long (up to 423 bp) recombinants can be produced and mosaics of the expression cassette via segmental gene conversion are typical, meaning that the possible repertoire of variants is enormous [63]. Entire replacement of the cassette with a silent gene has not been observed [63]. At times, “intermittent recombination events” occur, in which part of one silent gene is inserted into the expression cassette, followed by parental sequence and then more inserted DNA from a silent cassette [63]. Additional mutations in *vlsE* occur by single nucleotide changes, triplet repeats that allow for expansion or contraction, insertions/deletions, and “illegitimate recombination”, although the authors report that “some extent of sequence complementarity and alignment is needed to nucleate the recombination event” [63]. Despite all of the mechanisms for production of variability, rarely do they cause disruption of the *vlsE* reading frame, suggesting that natural selection maintains full functioning of this antigen.

An interesting question is what function is served by varying the VlsE antigen. Variant production occurs as early as four days after infection in immunocompetent mice [64] and antibodies for each expressed variant are produced in vivo in mice [65]. Any given infected individual typically has many simultaneous circulating variants [66]. A mutant strain lacking *vlsE* variant could not be maintained even in the white-footed mouse reservoir, was not acquired by the natural deer tick vector (because of low bacterial load in the host), but could induce a primary infection in the rodent [67]. Variant VslE is not required for infection in immunosuppressed mice [68] or for first-time infection of immunocompetent hosts [67]. Attempts to induce antigenic variation ex vivo are generally unsuccessful [63]. It also is possible that a variant VlsE strain might be capable of reinfecting a previously infected host, but this was shown not to be the case in mice, likely because antibodies against the other, invariant antigens prevented the new infection [69]. These data strongly suggest that immune evasion contributing to chronic infection is a key reason for VlsE to vary. This also suggests that the VlsE does not coat the other antigens and confer a protective effect. Antibody production to VlsE is considerable and T-cell independent, likely “confining” host adaptive immune response against other antigens. Unlike *B. hermsii*, there was not evidence for tissue tropism of different genetic strains of *B. burgdorferi*, suggesting that immune evasion was a more important force for evolution of varying antigens than tissue tropism [63].

### 1.6 *Neisseria gonorrhoeae* and *Neisseria meningitidis*

The pathogenic Neisserias (*N. gonorrhoeae* and *N. meningitidis*) are potentially epidemic, invasive, and fatal human pathogens. *N. meningitidis* is a nasopharyngeal commensal in up to 25% of healthy individuals but can develop into an epidemic pathogen, while gonococci colonize the urogenital tract and are sexually-transmitted bacterial pathogens. In both cases, epidemics typically represent invasion and spread of genetically clonal *Neisseria* strains; for the meningococci, capsular lineage A is responsible for huge outbreaks in Africa, and B and C in the industrialized world [70]. The means by which pathogenic and epidemic *Neisseria* strains emerge are not well understood but may include exposure of naïve human populations to new lineages or “unknown changes” yielding high-virulence clones [71].

A major adhesin for attachment to host epithelial cells is the filamentous, surface-exposed Type IV pilus which is required for host infectivity. Other potentially variant surface-exposed antigens include lipooligosaccharide, opacity-associated proteins (Opas), and a capsule. During passage and/or infection, cells can undergo phase shifts and become nonpiliated or the antigen can vary genetically but remain functional. Reportedly, pilus phase and antigenic variation in *Neisseria* spp. facilitate immune invasion and new host niche exploitation [71]. RecA-independent slipped strand mispairing can occur within the *pilC* genes [71] or pilin variation can occur through transformation from exogenous DNA [72]. However, antigen variants are also produced by RecA-dependent gene conversion from as many as 19 copies of the *pilS* locus (for pilus Silent) that lie within six chromosomal loci into the expressed *pilE* gene [73,74]. Silent copies lack promoters and ribosomal-binding sites but contain variable and conserved sequence on the 3′ end [71]. A *pilS* locus may contain a single gene copy or multiple, arranged in a tandem array [75]. The unidirectional recombination that produces *pilE* variants occurs at short (1–34 bp) areas of conserved sequence [76]. Similar to *B. burgdorferi*, recombinants into *pilE* can vary from very short to long, with insertions of 15 bp or less being common. Ten genes associated with DNA repair have already been shown to be involved in *pilE* switching, and to date, only the ruvA and ruvB involved in antigenic variation in *B. burgdorferi* appears to be common between the two pathogens [62]. Recently, a promoter was found 3′ of the *pilE* locus on the antisense strand of *N. meningitidis* which directs transcription of an anti-sense RNA and is expressed during particular bacterial growth phases and during salt stress [77]. This gives insight into a means for environmental influences on antigenic variation in this pathogen.

The Opa proteins (or protein II) are adhesins and have porin activity, with a multiple-copy family in the genome of approximately 11 copies [78]. In contrast to the pilin system consisting of a single expressed copy and multiple silent, defective copies, *Opa* gene copies are complete and each bacterial clone can express no, one, or two different Opa proteins [79]. *Opa* and *pilE* genes map close to each other in the genome [79]. The expression of Opa proteins regulates host cell tropism [75]. These proteins contain two surface-exposed hypervariable regions and variation is produced both by recombination and by horizontal transfer among clones [80]. However, one of the most important causes of reversible phase variation in Opas is via translational control. The 5′ signal peptide coding sequence contains variable numbers of tandem repeats of the sequence CTCTT: deletions and insertions of copies of this sequence can lead to a frameshift and mediate On/Off switching of Opas [81,82].

A mechanism to generate antigenic variation in *Neisseria* spp. bacteria is to relax the stringency of the mismatch correction system (MMC, sometimes termed mis-match repair, MMR) which repairs DNA mismatches and mutation following replication [76]. Mutator strains lacking functional genes for various components of the MMC can have up to 100 times higher rates of homologous recombination and lower thresholds for homology in order for recombination to proceed [83]. Interestingly, *N. gonorrhoeae* strains with defective *mutS* genes were less likely to have short mutations in the *pilE* but rather experience recombination events with longer > 200 bp tracts of replacement from *pilS* donors [84]. The MutS protein of the MMC binds to an upstream quartet of guanines (G4) to initiate the recombination and then serves to prevent homologous recombination [85,86]. However, use for different types of mutant MutS clarified that the increase in antigenic variation that occurs in *N. gonorrhoeae* is due to loss of mis-match repair, not altered G4 binding [85]. Defects in the MMC are over-represented in strains associated with invasive disease and epidemics [83]. *Neisseria* spp. also have contingency loci consisting of potentially large heterogeneous arrays of repetitive sequence tracts that facilitate transformation and recombination [87]. Horizontal transfer of genes with recombination has been recognized between meningococci, from commensal *Neisseria* strains to pathogens, and even between meningococci and gonococci [70].

*Neisseria* spp. vaccines have been produced for multiple strains targeting capsular polysaccharides, although antigenic variation and the impaired MMC in this genus complicate the problem. Particularly for serogroup B of *N. meningitidis* [88], additional problems are weak immunogenicity of some proteins and similarity to host antigens. A promising target has been the outer membrane protein factor H binding protein which host antibodies do target and candidate vaccines have already been developed for clinical trials. Unfortunately, the protein does show variability across geographical regions but there is also evidence for recombination sites and lateral transfer to vary the antigen, which may either reduce the value of a vaccine against this protein or at least require a collection of various vaccine types against all the various antigen strains [89].

### 1.7 *Treponema pallidum*

Syphilis is a sexually-transmitted disease caused by infection with the spirochete *T. pallidum*. Untreated disease can be chronic and result in primary chancres, disseminated rashes, neurological disorders, and death, likely associated with emergence of variant antigens that evade host immunity [90]. The *T. pallidum* repeat (*Tpr*) gene family consists of two subfamilies (I and II) and 12 members, of which the TprK antigen, presumably on the outer membrane, is most highly expressed [91]. The *TprK* gene undergoes segmental conversion to produce variant antigens [92]. There is a single *TprK* expression site consisting of conserved regions surrounding seven variable regions (V1–V7), each flanked by terminal 4 base pair repeat sequence. There also are internal repeat sequences. These 4 base pair repeats correspond to repeats in more than 50 donor sites flanking *TprD* and these serve whole or in pieces during segmental recombination to construct variant *TprK*. Each of the donor sites is relatively small and spans only part or whole of a single V region [92]. The differences observed within heterogeneous populations consist of base pair changes, insertions, and deletions [93].

Because the agent has not yet proven cultivable, rabbits are a commonly used animal model for syphilis [94]. Isolates that have been propagated in rabbits or obtained from human lesions are variable, although those bacteria from early chancres have less variation that those from older infections and bacteria show increased diversity over the course of passage [93]. Even within a single isolate, the amount of diversity varies across the variable regions [93]. These results are consistent with natural selection operating to promote variability and that the different V regions may be subject to different intensities of selection or at least vary in the rates of production of variation. There is good evidence that the emergence of a population of treponemes with variable TprK does occur in order to evade host immunity because antiserum raised against TprK opsonizes *T. pallidum* [95], immunosuppressed hosts accumulate lower levels of genetic diversity compared with competent hosts [96], and pre-immunized rabbits accumulated variation more quickly than control rabbits [96]. A recent study created clones of *T. pallidum* from the Chicago strain that did not vary in the *TprK* and used these in the rabbit model, documenting emergence of escape variants and the over-representation of the new variants in disseminated lesions, supporting earlier theories for the role of escape variants in immune evasion [94].

### 1.8 *Mycoplasma* Spp.

*Mycoplasma* spp. are typically mucosa-associated, cell wall-less bacteria with very small genomes and reduced protein coding capacity compared with other prokaryotes. Surface antigen variation in *Mycoplasma* spp. has been reported due to nucleotide insertion and deletions, DNA slippage, DNA rearrangement, site-specific recombination, gene conversion, and reciprocal recombination, in addition to transcriptional and other regulatory mechanisms [97,98]. *Mycoplasma genitalium* is a sexually transmitted bacteria that can cause urethritis, salpingitis, cervicitis, and pelvic inflammatory disease. Infection may last for several years [99]. The MgPa (or P140) and P110 proteins are associated with the attachment organelle and are encoded by *mgpB* and *mgpC*, respectively. Both genes are translated from a single expression site, of which there is only copy in the genome. However, approximately 4% of the genome contains MgPa repeat regions with variable homology to *mgpB* and *mgpC* [100]. Based on examination of the *M. genitalium* G-37^T^ genome, bacteria maintained in vitro, and observation of chronic infection, Iverson-Cabral documented extensive sequence variation within a single strain and that variation was produced through reciprocal recombination [97]. Within this genome, there are nine MgPa repeats and within most, segments homologous to *mspB* are interspersed with regions homologous to *mgpC*. The 5′ end, particularly of *mgpC*, matched to MgPa sequences and thus seems a target for homologous recombination. In vivo, during chronic infection, both genes varied extensively and independently. Results of sequencing of the expression site and MgPa regions were consistent with reciprocal recombination but not gene conversion, although some recombinants were asymmetrical. Occasionally, recombination appeared between MgPa repeat regions.

Ma et al. also showed that *mgpC* variants emerged by reciprocal recombination but also that variants emerged by gene conversion in a natural human chronic infection [101]. All except one of the MgPa repeats contained 3–5 “discrete minicassettes” with homology to multiple sites in *mgpB* and *mgpC* (referred to in this paper as MG191 and MG192). There also were conserved stretches of sequence in the MG192 variable region, which was interpreted as consistent with a site-specific recombination mechanism. In contrast, in vitro, mutants developed from large genomic deletions after *mgpB* and *mgpC* expression site cross-overs with MgPa regions [102]. The significance of these changes is not known as the work was done in vitro and non-attaching (avirulent) phenotypes were chosen for analysis [102].

*Mycoplasma pneumoniae*, a major cause of tracheobronchitis and community-acquired pneumonia, has a similar operon to *mgp*, containing the proteins P1 and the reading frames ORF4 and ORF6. RepMP fragments homologous to the P1 and ORF6 genes are dispersed and repeated 7–9 times in the genome, although little variation in expression of the P1 and ORF6 genes has been detected [103,104]. However, one novel P1 variant was detected with homology to two of the RepMP fragments, consistent with gene conversion [104]. A RecA homolog has been detected in both *M. genitalium* and *M. pneumoniae* which could be involved in recombination [105]. Additionally, characterization of the *Mpn*SSB protein of *M. pneumoniae* revealed its strong, selective single-stranded DNA binding activity and promotion of *Escherichia coli* recA-dependent recombination [106]

*Mycoplasma pulmonis*, the murine respiratory pathogen, has a cluster of genes called the *vsa* locus which encodes variable V1 antigens. There is a single functional expression site in one strain but multiple in another [107]. Nearby are silent *vsa* genes lacking functional promoters at the 5′ end but containing tandem repeats and recombination sites. Site-specific DNA inversions occur in which the promoter and the protein amino terminal coding DNA from the expression site is recombined into a previously silent gene [108]. The number of tandem repeats depends on the silent gene origin and varies through slipped-strand mispairing and illegitimate recombination [107]. A similar mechanism occurs in the related *Mycoplasma agalactiae vpma* locus [109].

Several avian mycoplasmas also show various methods of production of variants. Despite being closely related and having homologous antigen genes, the mechanisms used to induce variation are not the same. In both *Mycoplasma gallisepticum* and *Mycoplasma synoviae*, an important antigen is the variable lipoprotein hemagglutinin (VlhA) adhesin. Depending on the strain of *M. gallisepticum*, there may be up to 70 copies in the *vlhA* multiple-gene family [110] and a multiple-gene family also occurs for this gene in *M. synoviae* [111]. The gene families, while related, show important dissimilarities. In *M. gallisepticum*, tandemly repeated full-length genes, each with an intact ORF and promoter, are controlled by a variable trinucleotide repeat motif upstream of the promoter [110]. In contrast, only one intact gene exists for *M. synoviae*, but approximately 70 silent copies each lacking a promoter occur in tandem within the genome [112]. There are five different sites within the expressed gene into which pseudogenes may recombine to produce variants [111].

*Mycoplasma* spp. are fascinating for their incredibly parsimonious genomes, despite an apparent need for evading host immunity. *Mycoplasma* genomes are highly mutable with numerous mechanisms for mutation and recombination. For example, *Mycoplasmas* in general lack an SOS response and many repair proteins [113]. In theory, mycoplasmas may have high rates of homologous, site-specific, and illegitimate recombination in part to efficiently make use of large protein families encoded by minimal nucleotides [114]. Overall, the multiple and efficient mechanisms for recombination utilized by bacteria in this genus, including the rare reciprocal recombination, suggest a life history strategy that is distinctive among bacteria.

## 2 Evolution of Antigenic Variation

Major antigens vary genetically over the course of infection in a diversity of bacteria, particularly those with characteristically chronic infection, often in concert with cyclic adaptations of host immunity to expressed antigen variants. Unlike in viruses, mechanisms of antigenic variation in most bacteria involve larger DNA movement such as gene conversion or DNA rearrangement, with homologous recombination being perhaps the most prevalent. In contrast, some antigens vary due to point mutations or modified transcriptional regulation. The convergent evolution that promotes antigenic variation integrates various evolutionary forces: these include mutations underlying variant production; drift which could remove alleles especially early in infection or during life history phases in arthropod vectors (when the bacterial population size goes through a bottleneck); selection not only for any particular variant but also for the mechanism for the *production* of variants (i.e., selection for mutability); and overcoming negative selection against variant production.

### 2.1 The Origins of Variant Antigens

There is evidence that the origin of some antigens is from horizontal transfer, e.g., the *vlsE* gene has a much higher G:C content than the rest of the *B. burgdorferi* genome, suggesting an external source [115]. Presence of mobile elements among bacteria with antigenic variation varies: *Anaplasma* and *Ehrlichia* spp. lack prophages and transposable elements while *Wolbachia pipientis* has both phages and transposons [8,116]. The functions of the varying antigens are not thoroughly understood: some pathogens utilize the surface-exposed antigens for host cell interactions while the function of some antigens, such as those of *T. pallidum* is unknown.

Pathogens vary with respect to whether they express one or multiple antigens and how these antigens interact. For example, the outer membrane of *T. pallidum* reportedly has very few proteins and the TprK may be one of the few [117], while in contrast *Neisseria* spp. have Opa, pilin, and lipooligosaccharide antigens. In *B. burgdorferi*, there also are multiple antigens, so the fact that adaptive host responses to at least three invariant antigens fail to contain infection while the VlsE protein varies was enigmatic and led to the hypothesis that VlsE might physically cover the other antigens. This was not supported however by evidence that reinfection with strains expressing variant VlsE was not possible, suggesting that host immunity was directed against other antigens [69]. A revised theory is that the dominant antigen might manipulate host immunity during active infection, possibly through T-cell independent mechanisms that allow for futile antibody induction against the VlsE [69]. *Anaplasma* spp. may have multiple antigens, e.g., the Msp2 and Msp3 of *A. marginale*, but variation can occur in both. Thus evolution has not converged on a single strategy for expression of a single vs. multiple variant or invariant antigens.

### 2.2 Types of Mutation and Mutational Constraints

Across the prokaryotes, a diversity of mechanisms is used to generate diversity in antigens, including point mutations, DNA rearrangement, illegitimate recombination, reciprocal recombination, and by far the most common, homologous recombination. True illegitimate recombination may be uncommon; short, somewhat homologous tracts of DNA appear to be required to “nucleate” recombination of antigens in both *B. burgdorferi* and *Neisseria* spp. [63]. Duplication and recombination are common on *B. burgdorferi* linear plasmids, notably including the inverted repeats on the DHS [115]. Once DNA repeats are present, these repeats facilitate slipped-strand replication and/or recombination and these areas can become contingency loci [118]. Examples of this are the poly (dA–dT) tract at the *vmp* expression site of *B. hermsii* [43] and the repeat tracts that comprise at least 5% of the pathogenic *Neisseria*'s genomes [119]. While the frequency or probability of antigen switching could vary as a function of percent homology among conserved regions, this is not always the case, with a notable exception being *B. hermsii*, in which the probability of antigen switching differs as a function of distance from downstream and upstream homology regions [53].

The terminology among bacteria varies, but many species capable of varying antigens retain a library of unexpressed pseudogenes, silent genes, or cassettes that serve as material, after recombination into an expression site, for antigenic diversity. Large amounts of the genomes of *B. burgdorferi* and *A. phagocytophilum* are made up of pseudogenes, even though pseudogenes overall are uncommon in most bacteria [115]. The pattern of gene conversion among these bacteria differs: for *B. burgdorferi*, gene conversion into the *vlsE* cassette is “partial” and boundaries for recombination are within the gene per se, unlike in relapsing fever borrelias [61]. For *Anaplasma* spp., pseudogene fragments are inserted between conserved regions of the expression site. In contrast, even though *Ehrlichia* spp. can induce chronic infection and have a similar multiple-gene family encoding an immunogenic outer membrane protein, these genes are tandemly arranged full copies that appear to allow for antigenic variation though differential expression, not gene conversion. Variant bacterial antigens also differ as to the extent of the recipient gene that is replaced, i.e., essentially completely in the “cassette” system of *Anaplasma* spp. vs. the small fragments inserted for *T. pallidum* or variably sized pieces in *B. burgdorferi*. Even where an entire cassette can be shuffled into a recipient sequence, additional insertions or segmental gene conversion can produce mosaic antigens such as occurs in *A. marginale*. Segmental gene conversion and utilization of small donor sequences may allow for the bacteria to carry a lower pseudogene load, because longer donor DNA stretches are not required.

The location of silent gene copies may contribute to the mutability, e.g., in *A. phagocytophilum*, in which most copies are located near the putative origin of replication where multiple replication forks could increase recombination probabilities [8,9]. *Borrelia* spp. telomeres are reportedly “inherently recombinogenic” [120], increasing the likelihood of variant production in *B. hermsii* Vmps. No obvious pattern exists that pseudogenes are more likely to be inserted from a plasmid into a chromosomal expression locus, rather, *Anaplasma* spp. lack plasmids and the *Borrelia* spp. expression sites are on linear plasmids. In an unusual case, *opa* and *pilE* genes of the *Neisseria* spp. map together, which may reflect simply a recombinational hotspot but also a possible mechanism for efficient production of variation across antigens [121]. Pseudogene position and the differential probability of expression of particular pseudogenes could partially account for why there appears to be some programming in the chronological order of expression of alleles, as occurs in *A. marginale* and *B. hermsii*. Of course another reasonable explanation is that some clones in which variant antigens have been produced by recombination are relatively more fit, possibly due to differences in the functionality of antigen (outside its role in immune evasion), and that these clones would predominate early in infection, before host immune pressure mounts. There also is evidence in some bacteria that particular pseudogenes are more likely to be incorporated into an expression site compared to others, as in *Neisseria* spp. and *A. phagocytophilum* in which certain *pilS* or *msp2* alleles predominate [9,76]. Clearly this could be because of biochemical constraints such as chromosomal position, percent similarity of the conserved regions, or length of the flanking tracts vs. selective advantages of the particular alleles. For *A. phagocytophilum*, however, it is likely that differential fitness plays some role because usage of some pseudogenes appears to differ depending on which host it originates from.

It is not clear why the actual mechanisms of antigenic variation overwhelmingly represent homologous recombination/gene conversion. This does not appear to be a shared, derived character but rather has evolved independently multiple times. There are other mechanisms that produce variant antigens such as the reciprocal recombination that is found in *Mycoplasma* spp. and the homologous recombination in *Neisseria* spp. Even within the mycoplasmas, there are multiple mechanisms of antigenic variation which appear to have evolved independently [111]. Homologous recombination requires conserved tracts of DNA to anchor the reaction in both donor and recipient and primarily evolved as a mechanism to repair DNA after inaccurate replication and to incorporate genetic material in horizontal gene transfer between bacteria. Several different enzymes are involved in bacterial homologous recombination and, across the bacteria that utilize antigenic variation, there are differences in the availability of specific enzymes. For example, RecBCD is necessary for homologous recombination in *E. coli*, but *Anaplasma* spp. lack the RecBCD enzyme complex and instead vary antigens using RecF [6]. However, conserved DNA tracts flanking variable regions (that typically interact with host T and B-cells) are common. It is possible that mechanisms such as illegitimate recombination could be less reliable in producing functional but variant antigens that could contribute to evasion of host immunity.

### 2.3 Drift

Drift refers to the loss of genetic variability that occurs through random processes and is exacerbated at small sample sizes (e.g., during founder events). Bacteria vary considerably in the infectious dose needed to produce infection in the host, but in highly pathogenic species, this can be fairly low. Additionally, vector-borne pathogens commonly retain quite low copy numbers of bacteria in the invertebrate vector although replication may produce many copies at the time of transmission. The actual inoculum from the arthropod into the host may contain few bacteria. Where drift is relevant, it would be expected that early infections would involve relatively invariant bacteria and that genetic diversity of antigens would emerge over the course of chronic infection. In fact this has been observed in *T. pallidum* in which primary skin chancres do have invariant bacterial populations compared with bacteria from later-stage infections [93]. *Neisseria* strains from human epidemics are distinctly clonal compared with commensal strains, consistent with invasion of a new bacterial strain from a restricted population: in principle, over time these epidemic strains would likely accumulate genetic variability as well [83].

### 2.4 Natural Selection for Antigenic Variation

By far, the most commonly invoked explanation to account for the phenomenon of antigenic variation is evasion of host immunity in order to promote chronic infection, particularly in vector-borne and sexually-transmitted diseases [122]. Immune selection could also be invoked to show how antigen variation can facilitate reinfection or superinfection of host individuals with new genetic variants of a pathogen. However, it is also important to incorporate into theory the fact that antigens have other functions for their cells besides attracting host immunity. In many cases, phenotypes that vary could confer advantages for the pathogen such as allowing it to utilize diverse or varying host niches.

Immune evasion is at least a partial explanation for antigenic variation in all of the bacteria described in this paper, with the strength of the evidence dependent on studies showing concerted adaptive immune responses of the host with emergence of pathogen variants. Such natural selection is a form of frequency-dependent selection and was noted for *B. hermsii* where there is selection for rare variants [48]. Evidence for antigen variability conferring diverse tissue tropisms is mixed: variant *B. hermsii* and *A. phagocytophilum* strains exploit different hosts and tissues but this has not been shown for *B. burgdorferi*. This could be particularly relevant for *A. phagocytophilum* which infects a diversity of different host species [123]. Infections may be acute or chronic, depending on the host, revealing that evasion of host immunity is not always successful and suggesting that antigenic variation could serve some other function. The source of the very large number of pseudogenes in *A. phagocytophilum*, used in some hosts for antigenic variation, is an interesting question. The related *A. marginale* has far fewer pseudogenes yet is evolutionarily older [124], suggesting that an ancestral condition could have been fewer pseudogenes and the proliferation of pseudogenes has occurred more recently, possibly conferring the host breadth that characterizes *A. phagocytophilum* but not *A. marginale*. Exploitation of various host niches has also been proposed as a driver for antigenic variation in *M. pneumoniae*, as host antibodies did not appear to respond to the expression of emerging antigens [107] and for *M. hominis* [125].

Reinfection is possible in some but not all hosts with variant pathogens. For example, even strains of *B. burgdorferi* with distinctly different VlsE expression could not reinfect mice, likely because hosts already had developed strong immune responses to other, invariant antigens [69]. However, if antigenic variation does allow for reinfection or superinfection, then this could facilitate horizontal transmission of pathogen DNA between strains and promote rapid evolution.

### 2.5 Natural Selection for Hypermutability

In order to insure that new antigens will continue to be available over the course of chronic infection, some bacteria have evolved a state of hypermutability. This can be effected through physical construction of the genome (e.g., presence of anchoring repeats) such that recombination becomes more likely, dependence on faulty mutation–repair systems, or inaccurate replication machinery. There is evidence for hypermutability of genomes in *Borrelia* spp. in which large amounts of recombination, duplications, and pseudogenes suggest rapid evolution [115]. Some *Mycoplasma* spp. lack an SOS response and DNA repair proteins. Interestingly, the rate of variant production is considerably reduced in a recA deletion mutant of *M. gallisepticum*, suggesting a role for this repair protein in antigen variation [102]. Overall, recA was shown to have a minor role in general DNA repair of this pathogen, and thus these results tht recA is maintained in this highly reduced genome specifically for its ability to promote antigenic variation. Defects in the mismatch repair system induce hyper mutability in *Neisseria spp*.

### 2.6 Costs of Antigenic Variation: Natural Selection against Varying Antigens

One considerable benefit of high recombination rates in intracellular bacteria is to avoid “Muller's ratchet”, i.e., the accumulation of deleterious mutants [126]. However, it is important for the rate of mutation to be set at an intermediate level: very fast rates escape host control but risk producing too high a proportion of unfit progeny, but very slow rates fail to allow the pathogen to evade host immunity [127]. Some mechanisms for varying antigens could be riskier than others. For example reliance on slipped–strand recombination or illegitimate recombination could result in a overall genomic instability, producing not only less fit genes but potentially less fit genomes as well. Where gene conversion is used as a strategy for varying antigens, particularly if a repertoire of donor or pseudogenes is retained in the genome, a cost is incurred by the bacterium in order to repeatedly replicate an otherwise needlessly large genome. In general, bacterial genomes tend to evolve to be as small as possible: in intracellular bacteria, this pattern is exaggerated with extreme loss of unneeded genes [128]. However, at least transiently during evolution of a small genome, there may be an increase in the number of pseudogenes and mobile genetic elements. This pattern fits for the extremely small *Mycoplasma* spp. which generate variability using a diversity of mechanisms. A remarkable exception is *A. phagocytophilum*, an obligately intracellular pathogen which has an enormous genome consisting of more than 100 functional pseudogenes.

An obvious cost of using hypermutability to generate novel antigens is the risk of producing defective products from other genes as well, although strong purifying natural selection could occur on many genes to ensure that they don't vary. Purifying selection occurs even in some parts of antigen genes needed for functionality, e.g., in a *B. hermsii vsp* region corresponding to the signal peptide [48]. *A. marginale* Msp2 mosaics are selectively disadvantageous in immunologically naïve animals [10,129,130], theoretically because Msp2 proteins may have a porin function in addition to serving as an antigen, and immune-escape variants may have lower fitness as porins [13]. Even recombination targeting antigen genes incurs a cost if the new variants are less functional than the original, although this is difficult to assess for those bacteria for which the actual function of the antigen is poorly understood (e.g., TprK in *T. pallidum*). The pilus of *Neisseria* spp. is a very important virulence determinant and phase-shifted clones without pili are less fit *except* with respect to evading host immunity. It may be that dysfunctionally mutated *pilE* genes occur but that selection for functionality forces only some of these to persist in natural infections. However, the strength of the natural selection imposed by host immunity is considerable and in some cases could overcome the otherwise deleterious effects of the mutation.

Across the eubacteria, antigenic variation has emerged as a strategy to evade host immunity, while those same antigen molecules of have non-immune related functions as well, e.g., allowing the pathogen to exploit variable host niches. The specific mechanisms are not shared-derived characters although there is considerable convergent evolution and numerous commonalities reflecting considerations of natural selection and biochemical restraints. This review highlights the complexities of drivers of antigenic variation, in particular both those responding to immune stimuli but also extending evaluation to non-immune functions as well. A deeper understanding of the diversity of purpose and mechanisms of “antigenic variation” in bacteria will contribute to greater insight into bacterial pathogenesis, ecology and coevolution with hosts.

## References

[1] Palmer G., Bankhead T., Lukehart S. (2009). Nothing is permanent but change—antigenic variation in persistent bacterial pathogens. Cell Microbiol Epub.

[2] Vink C., Rudenko G., Seifert H.S. (2012). Microbial antigenic variation mediated by homologous DNA recombination. FEMS Microbiol Rev.

[3] Mahan S.M., Craig A., Scherf A. (2003). Antigenic variation in *Anaplasma marginale* and *Ehrlichia* (*Cowdria*) *ruminantium*. Antigenic variation.

[4] Park J., Kim K.J., Grab D.J., Dumler J.S. (2003). *Anaplasma phagocytophilum* major surface protein-2 (Msp2) forms multimeric complexes in the bacterial membrane. FEMS Microbiol Lett.

[5] Barbet A.F., Lundgren A., Alleman R., Stuen S., Bjöersdorff A. (2006). Structure of the expression site reveals extensive global diversity in MSP2/P44 variants of *Anaplasma phagocytophilum*. Infect Immun.

[6] Lin Q., Zhang C., Rikihisa Y. (2006). Analysis of involvement of the RecF pathway in p44 recombination in *Anaplasma phagocytophilum* and in *Escherichia coli* by using a plasmid carrying the p44 expression and p44 donor loci. Infect Immun.

[7] Granquist E.G., Stuen S., Crosby L., Lundgren A.M., Alleman A.R. (2010). Variant-specific and diminishing immune responses towards the highly variable MSP2 (P44) outer membrane protein of *Anaplasma phagocytophilum* during persistent infection in lambs. Vet Immunol Immunopathol.

[8] Dunning Hotopp J.C., Lin M., Madupu R., Crabtree J., Angiuoli S.V. (2006). Comparative genomics of emerging human ehrlichiosis agents. PLoS Genet.

[9] Foley J.E., Nieto N.C., Barbet A., Foley P. (2009). Antigen diversity in the parasitic bacterium *Anaplasma phagocytophium* arises from selectively-represented, spatially clustered functional pseudogenes. PLoS One.

[10] Barbet A.F., Lundgren A., Yi J., Rurangirwa F.R., Palmer G.H. (2000). Antigenic variation of *Anaplasma marginale* by expression of MSP2 mosaics. Infect Immun.

[11] Futse J.E., Brayton K.A., Nydam S., Palmer G.H. (2009). Generation of antigenic variants via gene conversion: evidence for recombination fitness selection at the locus level in *Anaplasma marginale*. Infect Immun.

[12] Brayton K.A., Kappmeyer L.S., Herndon D.R., Dark M.J., Tibbals D.L. (2005). Complete genome sequencing of *Anaplasma marginale* reveals that the surface is skewed to two superfamilies of outer membrane proteins. Proc Natl Acad Sci U S A.

[13] Brayton K.A., Palmer G., Azad A. (2012). Persistence and antigenic variation. Intracellular pathogens II: Rickettsiales.

[14] Futse J.E., Brayton K.A., Dark M.J., Knowles D.P., Palmer G.H. (2008). Superinfection as a driver of genomic diversification in antigenically variant pathogens. Proc Natl Acad Sci U S A.

[15] French D.M., McElwain T.F., McGuire T.C., Palmer G.H. (1998). Expression of *Anaplasma marginale* major surface protein 2 variants during persistent cyclic rickettsemia. Infect Immun.

[16] Eriks I.S., Palmer G.H., McGuire T.C., Allred D.R., Barbet A.F. (1989). Detection and quantitation of *Anaplasma marginale* in carrier cattle by using a nucleic acid probe. J Clin Microbiol.

[17] Brayton K.A., Meeus P.F., Barbet A.F., Palmer G.H. (2003). Simultaneous variation of the immunodominant outer membrane proteins, MSP2 and MSP3, during *Anaplasma marginale* persistence in vivo. Infect Immun.

[18] Palmer G.H., Brown W.C., Rurangirwa F.R. (2000). Antigenic variation in the persistence and transmission of the ehrlichia *Anaplasma marginale*. Microbes Infect.

[19] Ohashi N., Zhi N., Zhang Y., Rikihisa Y. (1998). Immunodominant major outer membrane proteins of *Ehrlichia chaffeensis* are encoded by a polymorphic multigene family. Infect Immun.

[20] Sulsona C., Mahan S.M., Barbet A. (1999). The map1 gene of *Cowdria ruminantium* is a member of a multigene family containing both conserved and variable genes. Biochem Biophys Res Commun.

[21] Ohashi N., Rikihisa Y., Unver A. (2001). Analysis of transcriptionally active gene clusters of major outer membrane protein multigene family in *Ehrlichia canis* and *E. chaffeensis*. Infect Immun.

[22] van Heerden H., Collins N.E., Brayton K.A., Rademeyer C., Allsopp B.A. (2004). Characterization of a major outer membrane protein multigene family in *Ehrlichia ruminantium*. Gene.

[23] Yu X., McBride J.W., Zhang X., Walker D.H. (2000). Characterization of the complete transcriptionally active *Ehrlichia chaffeensis* 28 kDa outer membrane protein multigene family. Gene.

[24] Frutos R., Viari A., Ferraz C., Morgat A., Eychenie S. (2006). Comparative genomic analysis of three strains of *Ehrlichia ruminantium* reveals an active process of genome size plasticity. J Bacteriol.

[25] Hughes A.L., French J.O. (2007). Homologous recombination and the pattern of nucleotide substitution in *Ehrlichia ruminantium*. Gene.

[26] Jiggins F.M., Hurst G.D., Yang Z. (2002). Host-symbiont conflicts: positive selection on an outer membrane protein of parasitic but not mutualistic Rickettsiaceae. Mol Biol Evol.

[27] Long S.W., Zhang X.F., Qi H., Standaert S., Walker D.H. (2002). Antigenic variation of *Ehrlichia chaffeensis* resulting from differential expression of the 28-kilodalton protein gene family. Infect Immun.

[28] Zhang X., Luo T., Keysary A., Baneth G., Miyashiro S. (2008). Genetic and antigenic diversities of major immunoreactive proteins in globally distributed *Ehrlichia canis* strains. Clin Vaccine Immunol.

[29] Werren J.H., Zhang W., Guo L.R. (1995). Evolution and phylogeny of *Wolbachia*: reproductive parasites of arthropods. Proc Biol Sci.

[30] McGraw E.A., O'Neill S.L. (2004). *Wolbachia pipientis*: intracellular infection and pathogenesis in *Drosophila*. Curr Opin Microbiol.

[31] Baldo L., Bordenstein S., Wernegreen J.J., Werren J.H. (2006). Widespread recombination throughout *Wolbachia* genomes. Mol Biol Evol.

[32] Braig H.R., Zhou W., Dobson S.L., O'Neill S.L. (1998). Cloning and characterization of a gene encoding the major surface protein of the bacterial endosymbiont *Wolbachia pipientis*. J Bacteriol.

[33] Baldo L., Lo N., Werren J.H. (2005). Mosaic nature of the *Wolbachia* surface protein. J Bacteriol.

[34] Baldo L., Bartos J.D., Werren J.H., Bazzocchi C., Casiraghi M. (2002). Different rates of nucleotide substitutions in *Wolbachia* endosymbionts of arthropods and nematodes: arms race or host shifts?. Parassitologia.

[35] Pham L.N., Dionne M.S., Shirasu-Hiza M., Schneider D.S. (2007). A specific primed immune response in *Drosophila* is dependent on phagocytes. PLoS Pathog.

[36] Barbour A.G. (1987). Immunobiology of relapsing fever. Contrib Microbiol Immunol.

[37] Alugupalli K.R., Gerstein R.M., Chen J., Szomolanyi-Tsuda E., Woodland R.T. (2003). The resolution of relapsing fever borreliosis requires IgM and is concurrent with expansion of B1b lymphocytes. J Immunol.

[38] Stoenner H.G., Dodd T., Larsen C. (1982). Antigenic variation of *Borrelia hermsii*. J Exp Med.

[39] Barbour A.G., Restrepo B.I. (2000). Antigenic variation in vector-borne pathogens. Emerg Infect Dis.

[40] Barbour A.G., Stoenner H.G., Herkowitz I., Simon M. (1985). Antigenic variation of *Borrelia hermsii*. Genome rearrangement.

[41] Restrepo B.I., Kitten T., Carter C.J., Infante D., Barbour A.G. (1992). Subtelomeric expression regions of *Borrelia hermsii* linear plasmids are highly polymorphic. Mol Microbiol.

[42] Kitten T., Barbour A.G. (1990). Juxtaposition of expressed variable antigen genes with a conserved telomere in the bacterium *Borrelia hermsii*. Proc Natl Acad Sci U S A.

[43] Barbour A.G., Burman N., Carter C.J., Kitten T., Bergstrom S. (1991). Variable antigen genes of the relapsing fever agent *Borrelia hermsii* are activated by promoter addition. Mol Microbiol.

[44] Barbour A., Carter C., Burman N., Freitag C., Garon C. (1991). Tandem insertion sequence-like elements define the expression site for variable antigen genes of *Borrelia hermsii*. Infect Immun.

[45] Sohaskey C.D., Zückert W.R., Barbour A.G. (1999). The extended promoters for two outer membrane lipoprotein genes of *Borrelia* spp. uniquely include a T-rich region. Mol Microbiol.

[46] Dai Q., Restrepo B.I., Porcella S.F., Raffel S.J., Schwan T.G. (2006). Antigenic variation by *Borrelia hermsii* occurs through recombination between extragenic repetitive elements on linear plasmids. Mol Microbiol.

[47] Kitten T., Barrera A.V., Barbour A.G. (1993). Intragenic recombination and a chimeric outer membrane protein in the relapsing fever agent *Borrelia hermsii*. J Bacteriol.

[48] Rich S.M., Sawyer S.A., Barbour A.G. (2001). Antigen polymorphism in *Borrelia hermsii*, a clonal pathogenic bacterium. Proc Natl Acad Sci U S A.

[49] Restrepo B.I., Carter C.J., Barbour A.G. (1994). Activation of a vmp pseudogene in *Borrelia hermsii*: an alternate mechanism of antigenic variation during relapsing fever. Mol Microbiol.

[50] Schwan T.G., Hinnebusch B.J. (1998). Bloodstream-versus tick-associated variants of a relapsing fever bacterium. Science.

[51] Raffel S.J., Battisti J.M., Fischer R.J., Schwan T.G. (2014). Inactivation of genes for antigenic variation in the relapsing fever spirochete *Borrelia hermsii* reduces infectivity in mice and transmission by ticks. PLoS Pathog.

[52] Pennington P.M., Allred C.D., West C.S., Alvarez R., Barbour A.G. (1997). Arthritis severity and spirochete burden are determined by serotype in the *Borrelia turicatae*-mouse model of Lyme disease. Infect Immun.

[53] Barbour A.G., Dai Q., Restrepo B.I., Stoenner H.G., Frank S.A. (2006). Pathogen escape from host immunity by a genome program for antigenic variation. Proc Natl Acad Sci U S A.

[54] Bacon R.M., Kugeler K.J., Mead P.S. (2008). Surveillance for Lyme disease—United States, 1992–2006. MMWR Surveill Summ.

[55] Brown R.N., Lane R., Dennis D.T., Goodman J.L., Dennis D.T., Sonenshine D.E. (2005). Geographic distributions of tick-borne diseases and their vectors. Tick-borne diseases of humans.

[56] Wormser G.P., Dattwyler R.J., Shapiro E.D., Halperin J.J., Steere A.C. (2006). The clinical assessment, treatment, and prevention of lyme disease, human granulocytic anaplasmosis, and babesiosis: clinical practice guidelines by the Infectious Diseases Society of America. Clin Infect Dis.

[57] Haake D.A. (2000). Spirochaetal lipoproteins and pathogenesis. Microbiol.

[58] Zhang J.R., Hardham J.M., Barbour A.G., Norris S.J. (1997). Antigenic variation in Lyme disease borreliae by promiscuous recombination of VMP-like sequence cassettes. Cell.

[59] Eicken C., Sharma V., Klabunde T., Lawrenz M.B., Hardham J.M. (2002). Crystal structure of Lyme disease variable surface antigen VlsE of *Borrelia burgdorferi*. J Biol Chem.

[60] Crother T.R., Champion C.I., Wu X.Y., Blanco D.R., Miller J.N. (2003). Antigenic composition of *Borrelia burgdorferi* during infection of SCID mice. Infect Immun.

[61] Zhang J.R., Norris S.J. (1998). Genetic variation of the *Borrelia burgdorferi* gene vlsE involves cassette-specific, segmental gene conversion. Infect Immun.

[62] Dresser A.R., Hardy P.-O., Chaconas G. (2009). Investigation of the genes involved in antigenic switching at the vlsE locus in *Borrelia burgdorferi*: an essential role for the RuvAB branch migrase. Plos Pathogens.

[63] Coutte L., Botkin D.J., Gao L., Norris S.J. (2009). Detailed analysis of sequence changes occurring during vlsE antigenic variation in the mouse model of *Borrelia burgdorferi* infection. PLoS Pathog.

[64] Zhang J.R., Norris S.J. (1998). Kinetics and in vivo induction of genetic variation of vlsE in *Borrelia burgdorferi*. Infect Immun.

[65] McDowell J.V., Sung S.Y., Hu L.T., Marconi R.T. (2002). Evidence that the variable regions of the central domain of VlsE are antigenic during infection with Lyme disease spirochetes. Infect Immun.

[66] Norris S.J. (2006). Antigenic variation with a twist—the *Borrelia* story. Mol Microbiol.

[67] Rogovskyy A.S., Casselli T., Tourand Y., Jones C.R., Owen J.P. (April 20, 2015). Evaluation of the importance of VlsE antigenic variation for the enzootic cycle of *Borrelia burgdorferi*. PLoS One.

[68] Labandeira-Rey M., Seshu J., Skare J.T. (2003). The absence of linear plasmid 25 or 28-1 of *Borrelia burgdorferi* dramatically alters the kinetics of experimental infection via distinct mechanisms. Infect Immun.

[69] Bankhead T., Chaconas G. (2007). The role of VlsE antigenic variation in the Lyme disease spirochete: persistence through a mechanism that differs from other pathogens. Mol Microbiol.

[70] Stephens D.S., Greenwood B., Brandtzaeg P. (2007). Epidemic meningitis, meningococcaemia, and *Neisseria meningitidis*. Lancet.

[71] Kline K.A., Sechman E.V., Skaar E.P., Seifert H.S. (2003). Recombination, repair and replication in the pathogenic Neisseriae: the 3 Rs of molecular genetics of two human-specific bacterial pathogens. Mol Microbiol.

[72] Gibbs C.P., Reimann B.Y., Schultz E., Kaufmann A., Haas R. (1989). Reassortment of pilin genes in *Neisseria gonorrhoeae* occurs by two distinct mechanisms. Nature.

[73] Koomey J.M., Falkow S. (1987). Cloning of the recA gene of *Neisseria gonorrhoeae* and construction of gonococcal recA mutants. J Bacteriol.

[74] Hamrick T.S., Dempsey J.A., Cohen M.S., Cannon J.G. (2001). Antigenic variation of gonococcal pilin expression in vivo: analysis of the strain FA1090 pilin repertoire and identification of the pilS gene copies recombining with pilE during experimental human infection. Microbiol.

[75] Hill S.A., Davies J.K. (2009). Pilin gene variation in *Neisseria gonorrhoeae*: reassessing the old paradigms. FEMS Microbiol Rev.

[76] Criss A.K., Kline K.A., Seifert H.S. (2005). The frequency and rate of pilin antigenic variation in *Neisseria gonorrhoeae*. Mol Microbiol.

[77] Tan F.Y., Wörmann M.E., Loh E., Tang C.M., Exley R.M. (2015). Characterization of a novel antisense RNA in the major pilin locus of *Neisseria meningitidis* influencing antigenic variation. J Bacteriol.

[78] Hobbs M.M., Seiler A., Achtman M., Cannon J.G. (1994). Microevolution within a clonal population of pathogenic bacteria: recombination, gene duplication and horizontal genetic exchange in the opa gene family of *Neisseria meningitidis*. Mol Microbiol.

[79] Stern A., Nickel P., Meyer T.F., So M. (1984). Opacity determinants of *Neisseria gonorrhoeae*: gene expression and chromosomal linkage to the gonococcal pilus gene. Cell.

[80] Smith J.M., Smith N.H., O'Rourke M., Spratt B.G. (1993). How clonal are bacteria?. Proc Natl Acad Sci U S A.

[81] Stern A., Brown M., Nickel P., Meyer T.F. (1986). Opacity genes in *Neisseria gonorrhoeae*: control of phase and antigenic variation. Cell.

[82] Murphy G.L., Connell T.D., Barritt D.S., Koomey M., Cannon J.G. (1989). Phase variation of gonococcal protein II: regulation of gene expression by slipped-strand mispairing of a repetitive DNA sequence. Cell.

[83] Richardson A.R., Yu Z., Popovic T., Stojiljkovic I. (2002). Mutator clones of *Neisseria meningitidis* in epidemic serogroup A disease. Proc Natl Acad Sci U S A.

[84] Criss A.K., Bonney K.M., Chang R.A., Duffin P.M., LeCuyer B.E. (2010). Mismatch correction modulates mutation frequency and pilus phase and antigenic variation in *Neisseria gonorrhoeae*. J Bacteriol.

[85] Rotman E., Seifert H.S. (2015). *Neisseria gonorrhoeae* MutS affects pilin antigenic variation through mismatch correction and not by pilE guanine quartet binding. J Bacteriol.

[86] Cahoon L.A., Seifert H.S. (2009). An alternative DNA structure is necessary for pilin antigenic variation in *Neisseria gonorrhoeae*. Science.

[87] Bayliss C.D., Field D., Moxon E.R. (2001). The simple sequence contingency loci of *Haemophilus influenzae* and *Neisseria meningitidis*. J Clin Invest.

[88] McNeil L.K., Zagursky R.J., Lin S.L., Murphy E., Zlotnick G.W. (2013). Role of factor H binding protein in *Neisseria meningitidis* virulence and its potential as a vaccine candidate to broadly protect against meningococcal disease. Microbiol Mol Biol Rev.

[89] Brehony C., Wilson D.J., Maiden M.C. (2009). Variation of the factor H-binding protein of *Neisseria meningitidis*. Microbiol.

[90] Gjestland T. (1955). The Oslo study of untreated syphilis; an epidemiologic investigation of the natural course of the syphilitic infection based upon a re-study of the Boeck-Bruusgaard material. Acta Derm Venereol.

[91] Centurion-Lara A., Castro C., Barrett L., Cameron C., Mostowfi M. (1999). *Treponema pallidum* major sheath protein homologue Tpr K is a target of opsonic antibody and the protective immune response. J Exp Med.

[92] Centurion-Lara A., LaFond R.E., Hevner K., Godornes C., Molini B.J. (2004). Gene conversion: a mechanism for generation of heterogeneity in the tprK gene of *Treponema pallidum* during infection. Mol Microbiol.

[93] LaFond R.E., Centurion-Lara A., Godornes C., Rompalo A.M., Van Voorhis W.C. (2003). Sequence diversity of *Treponema pallidum* subsp. *pallidum* tprK in human syphilis lesions and rabbit-propagated isolates. J Bacteriol.

[94] Reid T.B., Molini B.J., Fernandez M.C., Lukehart S.A. (2014). Antigenic variation of TprK facilitates development of secondary syphilis. Infect Immun.

[95] Centurion-Lara A., Godornes C., Castro C., Van Voorhis W.C., Lukehart S.A. (2000). The tprK gene is heterogeneous among *Treponema pallidum* strains and has multiple alleles. Infect Immun.

[96] Giacani L., Molini B.J., Kim E.Y., Godornes B.C., Leader B.T. (2010). Antigenic variation in *Treponema pallidum*: TprK sequence diversity accumulates in response to immune pressure during experimental syphilis. J Immunol.

[97] Iverson-Cabral S.L., Astete S.G., Cohen C.R., Totten P.A. (2007). mgpB and mgpC sequence diversity in *Mycoplasma genitalium* is generated by segmental reciprocal recombination with repetitive chromosomal sequences. Mol Microbiol.

[98] Citti C., Nouvel L.-X., Baranowski E. (2010). Phase and antigenic variation in mycoplasmas. Future Microbiol.

[99] Cohen C.R., Nosek M., Meier A., Astete S.G., Iverson-Cabral S. (2007). *Mycoplasma genitalium* infection and persistence in a cohort of female sex workers in Nairobi, Kenya. Sex Transm Dis.

[100] Fraser C.M., Gocayne J.D., White O., Adams M.D., Clayton R.A. (1995). The minimal gene complement of *Mycoplasma genitalium*. Science.

[101] Ma L., Jensen J.S., Myers L., Burnett J., Welch M. (2007). *Mycoplasma genitalium*: an efficient strategy to generate genetic variation from a minimal genome. Mol Microbiol.

[102] Burgos R., Pich O.Q., Ferrer-Navarro M., Baseman J.B., Querol E. (2006). *Mycoplasma genitalium* P140 and P110 cytadhesins are reciprocally stabilized and required for cell adhesion and terminal-organelle development. J Bacteriol.

[103] Ruland K., Himmelreich R., Herrmann R. (1994). Sequence divergence in the ORF6 gene of *Mycoplasma pneumonia*. J Bacteriol.

[104] Kenri T., Taniguchi R., Sasaki Y., Okazaki N., Narita M. (1999). Identification of a new variable sequence in the P1 cytadhesin gene of *Mycoplasma pneumoniae*: evidence for the generation of antigenic variation by DNA recombination between repetitive sequences. Infect Immun.

[105] Sluijter M., Spuesens E.B., Hartwig N.G., van Rossum A.M., Vink C. (2009). The *Mycoplasma pneumoniae* MPN490 and *Mycoplasma genitalium* MG339 genes encode RecA homologs that promote homologous DNA strand exchange. Infect Immun.

[106] Sluijter M., Hoogenboezem T., Hartwig N.G., Vink C. (2008). The *Mycoplasma pneumoniae* MPN229 gene encodes a protein that selectively binds single-stranded DNA and stimulates Recombinase A-mediated DNA strand exchange. BMC Microbiol.

[107] Rocha E.P., Blanchard A. (2002). Genomic repeats, genome plasticity and the dynamics of *Mycoplasma* evolution. Nucleic Acids Res.

[108] Bhugra B., Voelker L.L., Zou N., Yu H., Dybvig K. (1995). Mechanism of antigenic variation in *Mycoplasma pulmonis*: interwoven, site-specific DNA inversions. Mol Microbiol.

[109] Glew M.D., Papazisi L., Poumarat F., Bergonier D., Rosengarten R. (2000). Characterization of a multigene family undergoing high-frequency DNA rearrangements and coding for abundant variable surface proteins in *Mycoplasma agalactiae*. Infect Immun.

[110] Baseggio N., Glew M.D., Markham P.F., Whithear K.G., Browning G.F. (1996). Size and genomic location of the pMGA multigene family of *Mycoplasma gallisepticum*. Microbiol.

[111] Noormohammadi A.H. (2007). Role of phenotypic diversity in pathogenesis of avian mycoplasmosis. Avian Pathol.

[112] Allen J.L., Noormohammadi A.H., Browning G.F. (2005). The vlhA loci of *Mycoplasma synoviae* are confined to a restricted region of the genome. Microbiol.

[113] Razin S., Yogev D., Naot Y. (1998). Molecular biology and pathogenicity of mycoplasmas. Microbiol Mol Biol Rev.

[114] Dybvig K., Voelker L.L. (1996). Molecular biology of mycoplasmas. Annu Rev Microbiol.

[115] Casjens S., Palmer N., van Vugt R., Huang W.M., Stevenson B. (2000). A bacterial genome in flux: the twelve linear and nine circular extrachromosomal DNAs in an infectious isolate of the Lyme disease spirochete *Borrelia burgdorferi*. Mol Microbiol.

[116] Wu M., Sun L.V., Vamathevan J., Riegler M., Deboy R. (2004). Phylogenomics of the reproductive parasite *Wolbachia pipientis* wMel: a streamlined genome overrun by mobile genetic elements. PLoS Biol.

[117] Radolf J.D., Norgard M.V., Schulz W.W. (1989). Outer membrane ultrastructure explains the limited antigenicity of virulent *Treponema pallidum*. Proc Natl Acad Sci U S A.

[118] Moxon E., Rainey P., Nowak M., Lenski R. (1994). Adaptive evolution of highly mutable loci in pathogenic bacteria. Curr Biol.

[119] Snyder L.A., Butcher S.A., Saunders N.J. (2001). Comparative whole-genome analyses reveal over 100 putative phase-variable genes in the pathogenic *Neisseria* spp. Microbiol.

[120] Chaconas G., Stewart P.E., Tilly K., Bono J.L., Rosa P. (2001). Telomere resolution in the Lyme disease spirochete. EMBO J.

[121] Salit I.E., Blake M., Gotschlich E.C. (1980). Intra-strain heterogeneity of gonococcal pili is related to opacity colony variance. J Exp Med.

[122] Deitsch K.W., Lukehart S.A., Stringer J.R. (2009). Common strategies for antigenic variation by bacterial, fungal and protozoan pathogens. Nat Rev Microbiol.

[123] Foley J.E., Foley P., Brown R.N., Lane R.S., Dumler J.S. (2004). Ecology of granulocytic ehrlichiosis and Lyme disease in the western United States. J Vector Ecol.

[124] Foley J., Nieto N.C., Foley P., Teglas M.B. (2008). Co-phylogenetic analysis of *Anaplasma phagocytophilum* and its vectors, *Ixodes* spp. ticks. Exp Appl Acarol.

[125] Jensen L.T., Thorsen P., Moller B., Birkelund S., Christiansen G. (1998). Antigenic and genomic homogeneity of successive *Mycoplasma hominis* isolates. J Med Microbiol.

[126] Moran N.A. (1996). Accelerated evolution and Muller's rachet in endosymbiotic bacteria. Proc Natl Acad Sci U S A.

[127] Frank S.A., Barbour A.G. (2006). Within-host dynamics of antigenic variation. Infect Genet Evol.

[128] Moran N.A., Plague G.R. (2004). Genomic changes following host restriction in bacteria. Curr Opin Genet Dev.

[129] Palmer G.H., Futse J.E., Leverich C.K., Knowles D.P., Rurangirwa F.R. (2007). Selection for simple major surface protein 2 variants during *Anaplasma marginale* transmission to immunologically naive animals. Infect Immun.

[130] French D., Brown W., Palmer G. (1999). Emergence of *Anaplasma marginale* antigenic variants during persistent rickettsemia. Infect Immun.

